# Postoperative Pancreatitis Following Intraoperative Cholangiography: A Case Report

**DOI:** 10.7759/cureus.94822

**Published:** 2025-10-17

**Authors:** Chenyi Mao, Timothy Kenyon-Smith, Matthew J Shears

**Affiliations:** 1 Department of Medical Education, University of Melbourne, Melbourne, AUS; 2 Department of General Surgery, Northeast Health Wangaratta, Wangaratta, AUS

**Keywords:** intraoperative cholangiogram, laparoscopic cholecystectomy, pancreaticobiliary maljunction, pancreatitis, postoperative pancreatitis

## Abstract

Intraoperative cholangiography (IOC) is frequently performed during laparoscopic cholecystectomy to delineate biliary anatomy and detect choledocholithiasis. While generally safe, postoperative pancreatitis following IOC is an uncommon and under-recognized complication. We report the case of a 41-year-old male patient who presented with postprandial pain on a background of symptomatic gallstones. Preoperative blood tests, including liver function tests, were unremarkable, and ultrasound demonstrated gallstones with a non-dilated common bile duct (CBD). He underwent an uncomplicated laparoscopic cholecystectomy with IOC, which revealed contrast reflux into the pancreatic duct. The following day, he developed severe epigastric pain radiating to the back, nausea, vomiting, tachycardia, and low-grade fever. Blood tests showed elevated lipase (2000 U/L) and deranged liver function. Computed tomography (CT) cholangiogram confirmed acute interstitial pancreatitis without evidence of bile leak, choledocholithiasis, or pancreaticobiliary maljunction. He was managed with intravenous fluids, patient-controlled analgesia, and supportive care, and discharged home on day 4. This case highlights the importance of recognising contrast reflux as a possible precipitant of pancreatitis and the need for close postoperative monitoring.

## Introduction

Intraoperative cholangiography (IOC) is routinely performed during laparoscopic cholecystectomy to delineate biliary anatomy and detect choledocholithiasis, thereby reducing the risk of bile duct injury and retained stones [1]. The procedure involves cannulation of the cystic duct and injection of contrast to visualise the biliary tree. Although generally considered safe, complications such as bile duct injury, infection, and allergic reaction have been reported [2,3]. Postoperative pancreatitis following IOC is rare at 0.34%-0.69% [2,4] and often under-recognised. Although the exact mechanism is unknown, it is important to recognise those patients postoperatively, and we can give effective analgesia and bowel rest, and replace electrolytes. Here, we present a case of acute pancreatitis occurring after IOC in a patient with normal pancreaticobiliary anatomy to highlight this potential complication.

## Case presentation

A 41-year-old male patient developed acute pancreatitis subsequent to an emergency laparoscopic cholecystectomy with IOC. This follows a 24-hour period subsequent to a presentation to the emergency department, following a presentation characterised by postprandial pain, with a history of symptomatic gallstones. He had no relevant past medical history. Preoperative blood tests, including liver function tests, were unremarkable. His ultrasound demonstrated small mobile gallstones measuring from 4 to 5 mm, a 4 mm non-dilated common bile duct (CBD), and no evidence of cholecystitis.

**Figure 1:**
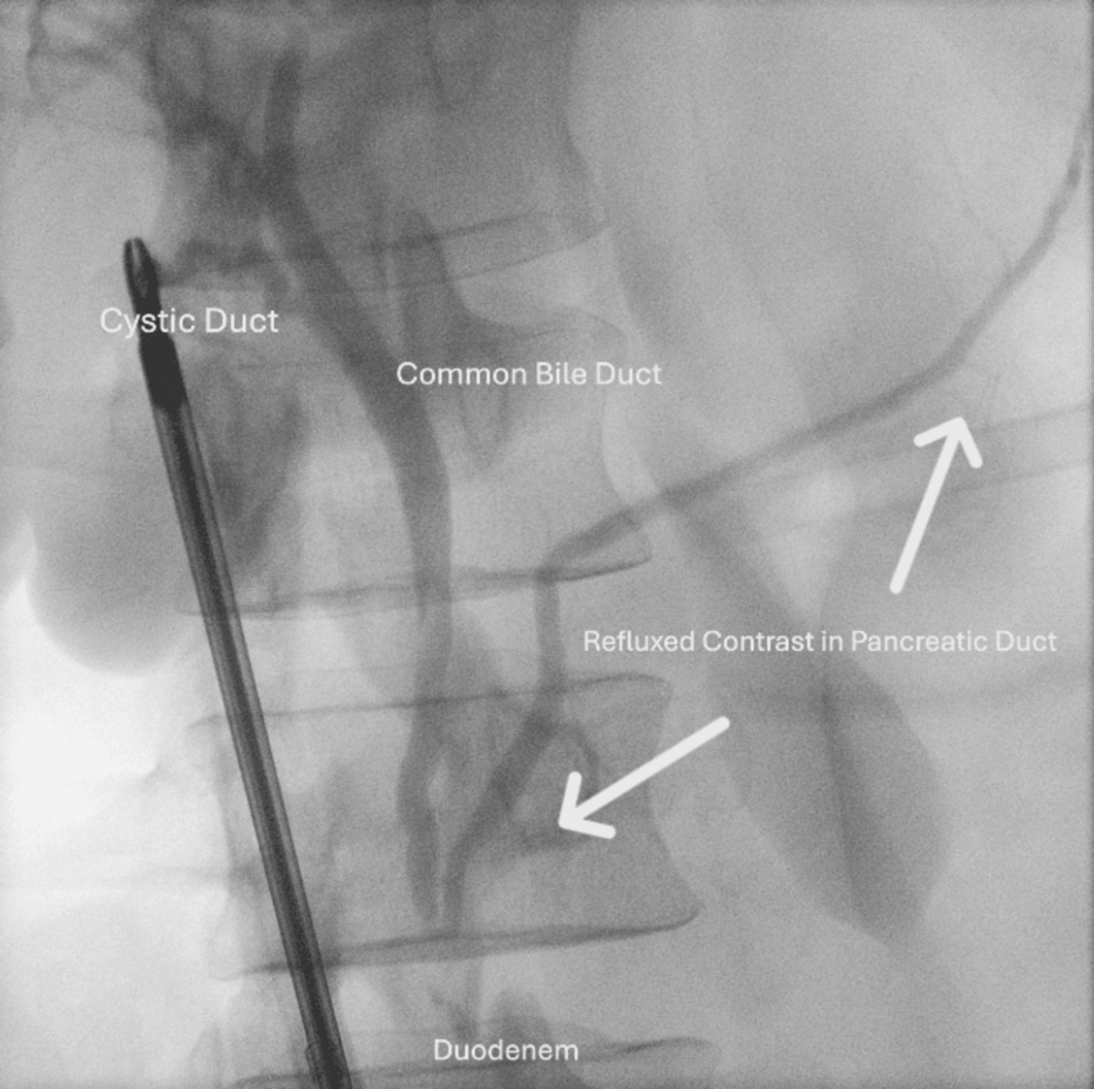
Intraoperative cholangiogram showing reflux of contrast medium (arrow) into the main pancreatic duct.

The laparoscopic cholecystectomy was straightforward. The only remarkable finding was the clear opacification of the pancreatic duct during the cholangiogram due to reflux of contrast (Figure 1). There were no filling defects identified on the cholangiogram.

**Figure 2:**
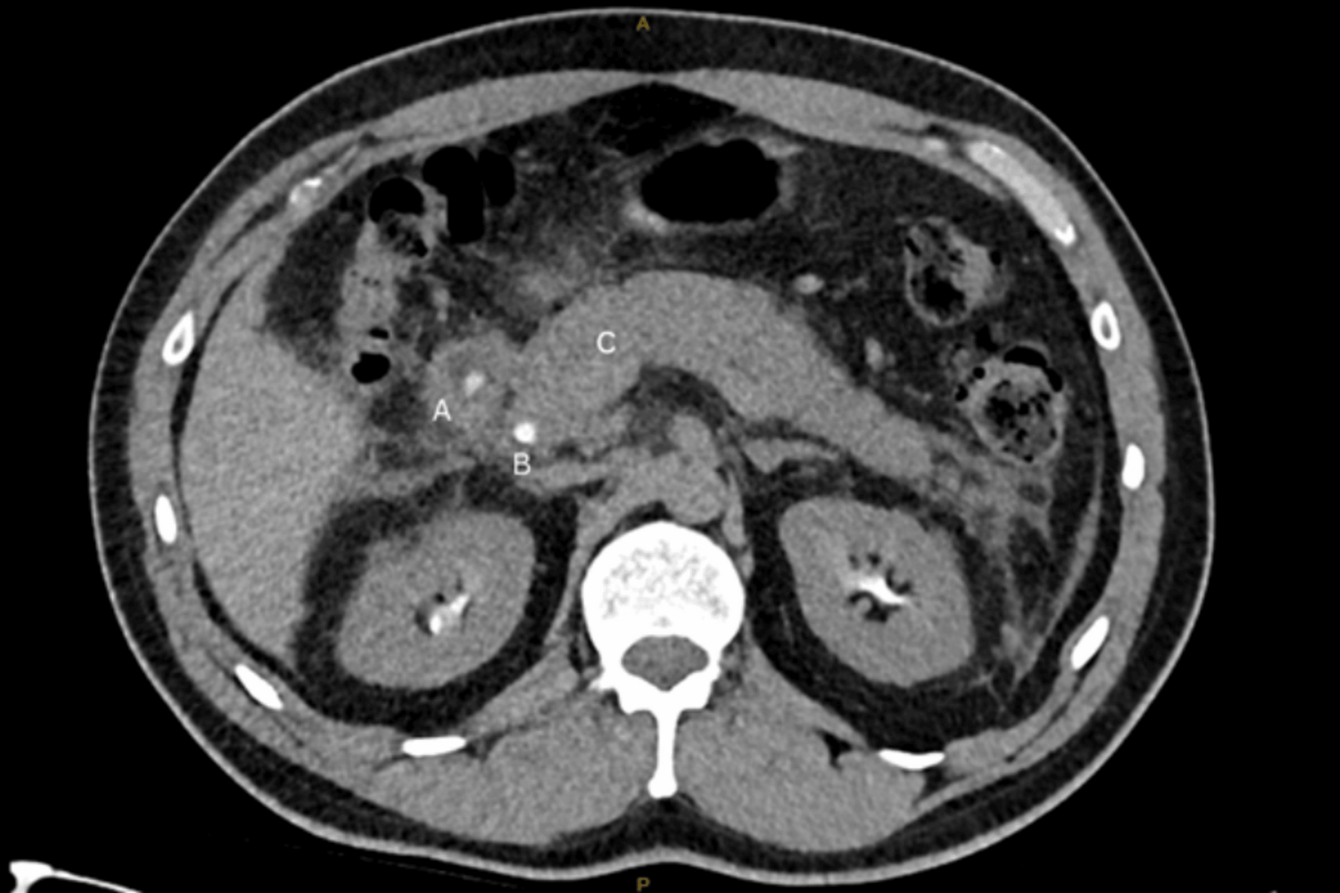
CT cholangiogram (axial view) on postoperative day 1 A: contrast in the duodenum, B: contrast in the common bile duct, C: mild fat stranding around the pancreas, suggestive of acute pancreatitis

The following day, he developed extreme epigastric pain radiating to the back with associated nausea and vomiting. His heart rate was 114 beats per minute, and he had a low-grade fever of 37.9 degrees Celsius, with other vital signs within normal parameters. His blood tests revealed elevated lipase and deranged liver function tests (Table 1). His computed tomography (CT) cholangiogram (Figure 2) subsequently demonstrated acute interstitial pancreatitis. There was no evidence of bile leakage or injury, pancreaticobiliary maljunction, or choledocholithiasis. 

**Table 1:** The patient's laboratory results

Test (unit)	Observed value	Normal range
Lipase (U/L)	2000	0–60
Bilirubin (µmol/L)	26	0–20
Alanine aminotransferase (U/L)	439	0–35
Aspartate aminotransferase (U/L)	115	0–35
Alkaline phosphatase (U/L)	104	30–110
Gamma-glutamyl transferase (U/L)	306	0–40

His moderate pancreatitis, classified according to the Revised Atlanta Classification [5], was managed with best supportive care, including fluid resuscitation and oxycodone for intravenous patient-controlled analgesia. He experienced no end-organ dysfunction and was discharged home four days later.

## Discussion

IOC is routinely performed during laparoscopic cholecystectomy to delineate biliary anatomy and identify choledocholithiasis. At our centre, Omnipaque™ contrast (Iohexol 240 mg/mL, GE HealthCare, Chicago, IL) is administered manually through a 20 mL syringe into a 4Fr cystic duct catheter. Although generally safe, postoperative pancreatitis following IOC is an under-recognised yet clinically important complication.

Identifying predictors of post-cholecystectomy pancreatitis remains crucial. The literature on its incidence and aetiology remains conflicting. A retrospective review from Western Australia by Sidiqi et al. reported a 0.69% incidence of post-cholecystectomy pancreatitis, with contrast reflux into the pancreatic duct significantly increasing the risk (p = 0.039) [2]. In contrast, a separate review by Morgan et al. found a similar incidence of 0.6%, but no statistically significant association between IOC and pancreatitis [3]. Reflux of contrast into the pancreatic duct during IOC has been proposed as a potential mechanism; however, the clinical implications of pancreatic reflux in patients without pancreaticobiliary maljunction are not clearly defined [6]. The relationship between IOC-related reflux and acute pancreatitis, therefore, remains debated and warrants further investigation [7].

Another proposed mechanism is microlithiasis. Microliths may induce functional obstruction of the sphincter of Oddi, resulting in papillitis or papillary spasm [8]. This appears unlikely in our case, given the normal preoperative bilirubin, absence of common bile duct dilatation on ultrasound, and a negative postoperative CT cholangiogram. Nevertheless, it is plausible that sludge in the distal CBD was disturbed and dislodged during IOC, with retrograde entry into the pancreatic duct, causing direct pancreatic injury. The subsequent rise in bilirubin may be explained by extrinsic compression of the bile duct from pancreatic inflammation and oedema.

## Conclusions

This case demonstrates that postoperative pancreatitis may occur following IOC due to contrast reflux into the pancreatic duct, even in patients with normal anatomy. Clinicians should be aware of this rare complication and monitor patients accordingly. Further research is warranted to better define this entity and to develop strategies for prevention and management.
